# Hydrogenium

**Published:** 1869-09

**Authors:** Robert Hunt


### HYDROGENIUM.

BY ROBERT HUNT, F.R.S.

The attention of experimental philosophers has, for some time past, been gradually drawn to the phenomena presented by the operation of some obscure force, or forces, ever active in the molecular interstices of matter. Under a variety of terms they have been explaining, or rather endeavoring to explain, peculiar attractions manifested by the surfaces of bodies, and assuming different conditions, according to the peculiarities belonging to the surfaces under examination. The force known as capillary attraction—whether exhibited in tubes or between plates of glass—is tolerably familiar to all, and the mechanical power shown by the fibre-tubes of cotton will have been tested by almost every intelligent schoolboy. The absorption of water by a lump of sugar or of chalk, and the "sucking up" of water by a sponge, is so common, that few stop to ask by what power the phenomenon is brought about. We are now, however beginning to discover that, in these, apparently, simple things, we may observe the opening of a door, disclosing a way, which promises to lead us to a knowledge of nature's most secret operations. The simple adhesion of water to a perfectly clean plate of glass, informs us, that a power resides on that surface; and, if we bring two such surfaces near together with a fluid between them, we see that the fluid is lifted against the gravitating influence of the whole Earth. In this we have hitherto detected a simple mechanical force only. Of late, however, M. E. Becquerel has informed us that this surface force has a power equal to the breaking up of strong chemical affinities. That, metallic solutions being employed, the metal is gradually separated from the solution and deposited in thin films upon the glass plates. In the fine fissures of green-stone rocks we often find films of native copper; and the films of gold in the cracks of the gold-bearing quartz are well known to the miner. These are doubtless due to the force resident on the surfaces of the rocks, in the same way as it is shown in action in M. E. Becquerel's experiment.

The influence of surface is discovered again in the ordinary process of filtration. It was shown by Dr. Hofmann and Mr. Witt, in their report on the water supply in London, that the water which passed through the filter beds of the water companies' reservoirs, was robbed of some portion of the salts held in solution. The late Dr. Normandy, when engaged in his experiments on the production of drinkable water from the sea, discovered that sea water was rendered free from salt, or nearly so, by being filtered through about thirty feet of siliceous sand or powdered glass. The removal of organic coloring matter from water, by passing through a few feet of earth, is another example of the same power in action. These phenomena are shown yet more strikingly, by charcoal. Hence its employment for purifying water, and its use for removing the annoyances arising from putrefactive fermentation. Experiments have shown that charcoal possesses the power, by virtue of its porosity, of condensing within itself many times its own volume of certain gases and vapors. This property is not peculiar to charcoal—all porous bodies exhibit it to a greater or less degree—but the power is strikingly manifested by this substance. It may be incidentally mentioned here, that Dr. Stenhouse has, by connecting a piece of charcoal with a voltaic battery, and plunging it into a solution of platinum, succeeded in coating all its interstitial spaces with a film of that metal. This is, in itself, another example of the surface action to which it is desired to draw attention. This platinized charcoal possesses all the powers of ordinary charcoal greatly exalted. It acts, indeed, as spongy platinum does, and not only condenses the gases escaping from putrid matter, but combines them with oxygen and slowly burns them away.

An instantaneous light lamp was common enough some years since. Hydrogen gas was produced, by a simple arrangement, by the oxidation of zinc in water, and stored in a bottle for use. When, by turning a stop-cock, a jet of hydrogen gas was projected upon a piece of spongy platinum, it was rapidly condensed and, at the same time, forced into combination with oxygen. The result of this was the production of heat sufficient to ignite the jet of hydrogen gas. Faraday showed how directly this depended on surface action. Taking a piece of perfectly clean platinum, he plunged it into a mixture of oxygen and hydrogen gases. These united to form water on the surface of the metal, and by the heat evolved, in this process, the metal became red hot.

It may appear too much to say that the solution of sugar or of salt in water is an analogous process to those which have been thus hastily and popularly described. A little attentive consideration will, however, carry conviction to the mind, that in the solution of a lump of sugar in water, we see the diffusion of it, through the interstitial spaces of the fluid, up to the point of saturation; when the solution-power ceases, and that it is a case of a similar nature to the solution of sulphuretted hydrogen in charcoal. Mr. Graham has, long since, beautifully shown the power of this surface force in water. Any one can repeat a simple experiment, and greatly interested will he be in watching the result. If to a solution of sulphate of copper some liquid ammonia is added, we produce that beautiful purple solution which marks the shop of a druggist. Fill a small bottle with this solution and placing a little bit of window-glass over the mouth of the bottle, lower it, by means of a string, into a confectioner's jar full of water. When it rests steadily at the bottom of the jar, carefully, with a rod, strike off the glass cover from the bottle. The water and the ammonia-sulphate of copper are in contact, but they do not mix. Gradually it will be observed that the purple solution loses color, becoming a pale blue. The chemical combination has been overthrown—the ammonia has left the sulphate of copper and diffused itself through the water. In a similar manner, yet more powerful chemical combinations may be broken up.

We are acquainted with other phenomena, in which modified conditions of the force which we have been considering are strikingly shown. Exosmose and endosmose—or, as Mr. Graham terms it, Osmose Force—exhibits phenomena of a peculiar character, yet a cautious examination appears to lead to the conclusion that there is little essential difference between it and the forms of force which have been described. A porous tile, a wall of clay, a piece of animal membrane, dividing two fluids, differing but slightly in their character—say, for example, sugar and water—shall be on one side of the partition, and water only on the other. Porosity immediately begins its work: the solid substance in solution (this mode of expression can scarcely be avoided, but the substance in solution does not exist in the solid state) passes through in one direction while a little of the purer fluid passes through in the other direction. Flowing in and flowing out goes on until all the sugar, or other substance, leaves its own cell and settles itself in the other. By this process numerous chemical decompositions can be effected, as in the cases already cited. In each and all of these phenomena, it is tolerably certain that we are dealing with an obscure, but a most energetic force, possessing more resemblance to gravitation than any other known power, but distinguished from it by broad lines of difference. In gravitation we discover a power acting, irresistibly, amongst the particles of matter, drawing all to a mathematical center, while, at the same time, we detect an influence—is it diffusive?—which binds mass to mass in space and regulates the motions of worlds. In the surface force under consideration we find a power acting in perfect independence of gravitation—often in opposition to it; but it is a caved giant, whose power is limited to the cave in which it dwells.

Pursuing a series of investigations, all of them being remarkable examples of experimental induction, and which may be regarded as originating in the more simple phenomena referred to, Mr. Graham was led to the discovery that certain metals not only absorbed some of the gases, and especially Hydrogen, but that they retained those metals, or as the discoverer termed it "*occluded*"* them. When iron or platinum or palladium in a state of tolerable purity—whether in the form of sponge, or aggregated by hammering—is heated, and allowed to cool slowly and completely in a hydrogen atmosphere, those metals are found to have absorbed many times their volume of the gas, and to hold it in a state of "*occlusion*" for any length of time; until, indeed, it is dispelled by heat. It was the discovery of this fact, and the examination of meteoric iron, which led to the remarkable discovery that these meteoric masses must have passed through—and indeed cooled in—an atmosphere of hydrogen gas. Mr. Graham advanced from this point to a knowledge of a new method of charging metals with hydrogen at low temperatures. When a plate of zinc is placed in dilute sulphuric acid, hydrogen gas is liberated from the water by the oxidation of the metal, and it is evolved from the surface of the zinc, but no hydrogen is occluded. Mr. Graham remarks, " a negative result was to be expected from

---

* *Occlusion* is a good old English word, signifying to 'shut up,' which had fallen out of use, until Mr. Graham restored it as a scientific term.

the crystalline structure of zinc." We are disposed to ask why crystalline structure should interfere with this power of retention? If, however, a thin plate of palladium is immersed in the same diluted acid, and brought into metallic contact with the zinc, the hydrogen is transferred to its surface, and the gas is largely absorbed. The charge taken up in an hour by a palladium plate, rather thick, at 12° amounted to 173 times its volume.

"The absorption of hydrogen was still more obvious when the palladium plate was constituted the negative electrode, in acidulated water, to a Bunsen battery of six cells. The evolution of oxygen gas at the positive electrode continuing copious, the effervescence at the negative electrode was entirely suspended for the first twenty seconds, in consequence of the hydrogen being occluded by the palladium. The final absorption amounted to 200 volumes."

The hydrogen enters the palladium and no doubt pervades the whole mass of the metal, but it exhibits no disposition to leave the metal, and escape into a vacuum, at the temperature of its absorption. Pieces of palladium charged with hydrogen have been sealed up in exhausted glass tubes. After two months the glass has been broken under mercury, and the vacuum found perfect, no hydrogen having vaporised in the cold, but on the application of heat 333 volumes of gas were evolved from the metal. Another experiment was of a very striking character. A hollow palladium cylinder was made the negative electrode in an acid fluid, while the closed cavity of the cylinder was kept exhausted by means of a Sprengel aspirator. No hydrogen whatever passed into the vacuous cavity in several hours, although the gas was no doubt abundantly absorbed by the outer surface of the cylinder, and pervaded the metal throughout.

It appears that when hydrogen is absorbed by the metal palladium, the volatility of the gas may be entirely suppressed; and hydrogen may be largely present in metals without exhibiting any sensible tension at low temperatures. "*Occluded hydrogen is certainly no longer a gas, whatever may be thought of its physical conditions.*"

It has often been maintained on chemical grounds that hydrogen gas—the lightest body in nature—is the vapor of a highly volatile metal. Sir Humphry Davy and others have drawn attention, from time to time, to certain conditions which appeared to connect hydrogen with the metals, and now the results obtained by the Master of the Mint appear to confirm those views. Mr. Graham remarks: "The idea forces itself upon the mind that palladium, with its occluded hydrogen, is simply an alloy of this volatile metal, in which the volatility of the one element is restrained by its union with the other, and which owes its metallic aspect equally to both constituents." The following brief statements of the conditions of palladium—and of palladium charged with hydrogen—will elucidate this point.

It should be stated, in the first place, that palladium in the state of thin films, as thrown down from a solution of the chloride by a voltaic batter, when heated to 100° in hydrogen, and allowed to cool slowly for an hour in the same gas, was found to occlude 982·14 volumes of the hydrogen. This is the largest absorption of hydrogen which has been observed, and certainly it is not a little remarkable to find a dense body, such as the metal palladium is, absorbing and retaining nearly one thousand times its volume of so light a body as hydrogen is. The density of palladium when charged with eight or nine hundred times its volume of hydrogen gas is perceptibly lowered. A palladium wire before exposure measured 609·144 millims (23·982 inches). This wire received a charge of hydrogen amounting to 936 times its volume, and increased in length 9·779 millims (or 0·385 inches); it measuring, when charged, 618·923 millims. The density of the charged wire is reduced from 12·3 to 11·79. The expulsion of hydrogen from the wire however caused, is attended with an extraordinary contraction of the latter On expelling the hydrogen by a moderate heat, the wire not only receded to its original length, but fell as much below that zero as it had previously risen above it. That a very remarkable change is produced in the palladium by the absorption of the hydrogen is shown by the manner in which it burns. A wire so charged with hydrogen, if rubbed with the powder of magnesia (to make the flame luminous), burns like a waxed thread when ignited in the flame of a lamp. It has been proved that the tenacity of palladium is altered by the occlusion of hydrogen. The tenacity of palladium wire being 100, the tenacity of palladium and hydrogen was found to be 81·29

Dr. Faraday determined, by many experiments, that palladium is "feebly but truly magnetic," and he placed this element at the head of what are now called *paramagnetic metals.* The experiments of Mr. Graham show that, with occluded hydrogen, palladium becomes so magnetic that it must be allowed to rise out of the paramagnetic class, and to take place in the strictly magnetic group with iron, nickel, cobalt, chromium, and manganese. Many chemical peculiarities distinguish this compound from ordinary palladium. The conclusions which appear to flow from this enquiry are, that in palladium fully charged with hydrogen there exists a compound of palladium and hydrogen in a proportion which may approach to equal equivalents. The charged palladium is represented by weight as

| | | |
|---|---|---|
| Palladium . . | 1·0020 grm . | 99·277 |
| Hydrogen . . | 0·0073 grm - | ·723 |
| | | 100 000 |

It is in the proportion of one equivalent of palladium to 0·772 equivalent of hydrogen H=1, Pd=106·5. The evidence is therefore strong that a true alloy is produced, and to this alloy the name of *Hydrogenium* has been given.

In this alloy hydrogen appears to be reduced to the metallic state, and the great problem of the chemist, as it regarded the physical condition of hydrogen, is satisfactorily solved. The magnetic character of this alloy may have its bearing upon the appearance of hydrogenium in meteoric iron, in association with certain other magnetic elements.

The absorption of hydrogen by palladium is a striking fact. That this gas is absorbed by platinum and by iron has also been proved. The occluded hydrogen found in meteorites points to a condition in space, upon which we can only obscurely speculate. Spectrum analysis is teaching us that this element—hydrogen—forms an important constituent of the nebulous groups and comentary films. The examination of surface forces instructs us that the element which, oxidised, becomes water, and which, in its combinations with carbon, plays so important a part in the animal and vegetable economy, is no less essential as an agent modifying the conditions of the mineral world. From the study of little things—the solution of sugar, the absorption of water by a sponge—we are advanced to the discovery of truths which bear on the mysteries of molecular structure, and on the constitution of worlds in space.

